# Forward Genetics Reveals a *gatC-gatA* Fusion Polypeptide Causes Mistranslation and Rifampicin Tolerance in *Mycobacterium smegmatis*

**DOI:** 10.3389/fmicb.2020.577756

**Published:** 2020-09-24

**Authors:** Rong-Jun Cai, Hong-Wei Su, Yang-Yang Li, Babak Javid

**Affiliations:** ^1^Centre for Global Health and Infectious Diseases, Collaborative Innovation Centre for the Diagnosis and Treatment of Infectious Diseases, Tsinghua University School of Medicine, Beijing, China; ^2^Beijing Advanced Innovation Center in Structural Biology, Beijing, China; ^3^Division of Experimental Medicine, University of California, San Francisco, San Francisco, CA, United States

**Keywords:** mistranslation, GatCAB, amidotransferase, *Mycobacterium*, antibiotic tolerance, persisters

## Abstract

Most bacteria, including mycobacteria, utilize a two-step indirect tRNA aminoacylation pathway to generate correctly aminoacylated glutaminyl and asparaginyl tRNAs. This involves an initial step in which a non-discriminatory aminoacyl tRNA synthetase misacylates the tRNA, followed by a second step in which the essential amidotransferase, GatCAB, amidates the misacylated tRNA to its correct, cognate form. It had been previously demonstrated that mutations in *gatA* can mediate increased error rates specifically of glutamine to glutamate or asparagine to aspartate in protein synthesis. However, the role of mutations in *gatB* or *gatC* in mediating mistranslation are unknown. Here, we applied a forward genetic screen to enrich for mistranslating mutants of *Mycobacterium smegmatis*. The majority (57/67) of mutants had mutations in one of the *gatCAB* genes. Intriguingly, the most common mutation identified was an insertion in the 3′ of *gatC*, abolishing its stop codon, and resulting in a fused GatC-GatA polypeptide. Modeling the effect of the fusion on GatCAB structure suggested a disruption of the interaction of GatB with the CCA-tail of the misacylated tRNA, suggesting a potential mechanism by which this mutation may mediate increased translational errors. Furthermore, we confirm that the majority of mutations in *gatCAB* that result in increased mistranslation also cause increased tolerance to rifampicin, although there was not a perfect correlation between mistranslation rates and degree of tolerance. Overall, our study identifies that mutations in all three *gatCAB* genes can mediate adaptive mistranslation and that mycobacteria are extremely tolerant to perturbation in the indirect tRNA aminoacylation pathway.

## Introduction

The vast majority of bacteria lack the specific aminoacyl tRNA synthetases (aaRSs) coding for glutaminyl-tRNA (GlnRS) and/or asparaginyl-tRNA (AsnRS) synthetases or both (Curnow et al., 1997; Sheppard and Soll, 2008). The exceptions are some gamma proteobacteria of which *Escherichia coli* is the most notable example – which have the full complement of 20 aaRSs (Sheppard and Soll, 2008). In organisms or organelles lacking GlnRS and/or AsnRS, a two-step indirect aminoacylation pathway is utilized to correctly aminoacylate tRNA^Gln^ and tRNA^Asn^. In the first step, a non-discriminatory glutamyl-tRNA (ND-GluRS) or aspartyl-tRNA (ND-AspRS) aminoacyl-tRNA synthetase physiologically misacylates tRNA^Gln^ to Glu-tRNA^Gln^ and tRNA^Asn^ to Asp-tRNA^Asn^, respectively. In both cases, the misacylated tRNA complex is specifically recognized by the products encoded by the essential heterotrimeric amidotransferase genes *gatCAB*. Using glutamine as an amide donor, GatCAB amidates Glu-tRNA^Gln^ to the cognate Gln-tRNA^Gln^ and Asp-tRNA^Asn^ to Asn-tRNA^Asn^, respectively (Curnow et al., 1997). GatA acts as an amidase: liberating ammonia from glutamine, whereby GatB specifically recognizes the misacylated tRNA complexes and subsequently performs the amidotransferase reaction. There is no known enzymatic function for the smallest subunit, GatC, which is thought to act as a stabilizing scaffold for the complex (Suzuki et al., 2015). In most bacteria, *gatC, gatA* and *gatB* are encoded in a single operon, however, in mycobacteria, *gatC* and *gatA* are in one operon, and the gene phosphofructokinase and *gatB* are in a second, immediately downstream operon (Cole et al., 1998; Sheppard and Soll, 2008). Of note, as is common in many bacterial operonic genes, in *M. smegmatis* there is an overlap between the 3′ end of *gatC* (encoding the termination codon TGA) and the start codon (ATG) of *gatA*.

We had previously performed a forward genetic screen to identify molecular mechanisms underlying the very high rates of asparagine to aspartate mistranslation previously demonstrated in *Mycobacterium smegmatis* (Javid et al., 2014; Su et al., 2016). We identified mutations in mycobacterial *gatA* that caused increased rates of mistranslation (Su et al., 2016), and showed that mutations in *gatA* identified from clinical isolates of *M. tuberculosis* also caused mistranslation, and importantly, tolerance to the antibiotic rifampicin (Su et al., 2016). This was the first identification of naturally occurring mutations from clinical bacterial isolates that supported a role for “adaptive mistranslation” (De Pouplana et al., 2014; Rathnayake et al., 2017). However, the potential roles, if any, of mutations in *gatC* and *gatB* in mediating mistranslation – and rifampicin tolerance – is completely unknown. Our prior screen involved an extremely stringent selection followed by screen strategy, designed to identify mutations that would cause only very high rates of mistranslation (Su et al., 2016). We hypothesized that the prior strategy may have missed mutations that would result in less extreme phenotypes. We therefore performed a modified forward genetic screen adapted from our prior work that would enrich for mutations causing moderate rates of mistranslation. We identified mutations in *gatC*, *gatA*, and *gatB* that caused both specific mistranslation and rifampicin tolerance. Of note, the most frequently identified mutation; in the 3′ end of *gatC*, resulted in the abolition of the *gatC* termination codon, resulting in a GatC-GatA fused protein. Our results suggest that mutations in all three constituent members of *gatCAB* may result in adaptive mistranslation, and that alteration of the native conformation of the essential gene products GatC and GatA results in viable bacteria, but with altered translational fidelity.

## Materials and Methods

### Bacterial Strains and Culture

Wild-type *Mycobacterium smegmatis* mc^2^-155 (ATCC) was cultured in Middlebrook 7H9 liquid broth supplemented with: 10% ADS (albumin-dextrose-salt), 0.2% glycerol and 0.05% Tween-80, or plated on LB (Lennox) agar. *E. coli* DH5α (CW Biotech) was used for cloning. Antibiotic concentrations for *M. smegmatis* were: hygromycin 40 μg ml^–1^, kanamycin 20 μg ml^–1^, zeocin 25 μg ml^–1^. Antibiotic concentrations for *E. coli* were: 150 μg ml^–1^ hygromycin, kanamycin 50 μg ml^–1^, zeocin 50 μg ml^–1^. All strains were grown at 37°C unless otherwise indicated.

### Selection for High Mistranslating *M. smegmatis*

Briefly, we followed a similar strategy as previously described (Su et al., 2016) with minor modifications. A strain of wild-type (WT) *M. smegmatis* expressing the mutated kanamycin kinase gene *aph* (either Aph-D214N or Aph-D214V) on an episomal plasmid pJW3-Aph (Wong et al., 2013; Javid et al., 2014) was grown overnight to stationary phase. Approximately 10^7^ or 10^8^ colony forming units (CFU) of each strain were plated onto each of 5 LB-agar plates each containing 10 μg/ml or 15 μg/ml kanamycin, respectively, from 2 independent cultures. Concentrations of kanamycin were chosen in pilot experiments that were just sufficiently high to suppress growth of *M. smegmatis*-pJW3-Aph-D214V but not *M. smegmatis*-pJW3-Aph-D214N, since rates of asparagine to aspartate but not valine to aspartate translational errors are naturally high in mycobacteria (Javid et al., 2014; Leng et al., 2015; Su et al., 2016), but not so high as to only select for *de novo* kanamycin resistance (Su et al., 2016). Colonies of 67 survivors, representing the independent cultures and both antibiotic selection concentrations were picked, patched and grown in small volume cultures. The *gatCA* and *gatB* genes of crude DNA extract from the strains was amplified by PCR and sent for standard Sanger sequencing. The pJW3 plasmid was cured by growth of strains to late stationary phase in the absence of antibiotics and patching onto LB-agar and LB-agar-zeocin. Colonies of strains cured of the plasmid (i.e., zeocin-sensitive) were used for downstream experiments. If the plasmid was not cured on the first passage for any one strain, the process was repeated a second time, by which time all strains had been cured.

### Western Blot

Mycobacteria were lysed by bead-beating in 25 mM Tris–HCl (pH 7.6), 100 mM NaCl and 1 mM EDTA with protease inhibitor (Roche). Polyclonal rabbit serum raised against a peptide from mycobacterial GatA was used as before (Su et al., 2016). For lane loading controls, a monoclonal antibody against EF-Tu (clone 900, AbCam) was used. SDS-PAGE and Western blotting were performed as previously described (Su et al., 2016; Zhang et al., 2019).

### Relative Mistranslation Rate Assay

This was performed as previously described (Chen et al., 2019). The 12 strains were transformed with plasmids expressing GFP (integrated) and secreted Nluc luciferase (episomal plasmid) in two steps (Chaudhuri et al., 2018) – both GFP and Nluc were under control of a tetracycline-inducible promoter. Strains were grown overnight until OD_600_ ∼ 3. Anhydrotetracycline (ATc) was added to a final concentration of 50 ng/ml to independent cultures and replicates plated in 96-well plates overnight and cultured with shaking at 37°C. The following day, 80 μL of each replicate was transferred to black, flat bottom 96-well plates (Corning #CLS3925) for GFP measurement using a Fluoroskan Ascent FL Microplate Fluorometer and Luminometer. The original culture plates were spun for 10 min to pellet bacteria and 40 μL of supernatant, which contained secreted Nluc luciferase was transferred from each well to white, flat bottom 96-well plates (Corning #CLS3922) for luminescence reading (using the Nano-Glo kit, Promega) and using the same instrument and 1000 ms integration time. Relative mistranslation rates were calculated as previously described (Chen et al., 2019) by correcting Nluc activity to GFP florescence.

### Molecular Structure Modeling

The model of the GatC-GatA/GatB fusion heterodimer (and wild-type GatCAB heterotrimer) were constructed using Rosetta (Song et al., 2013). Initially, for modeling the wild-type enzyme, the solved structure of *Staphylococcus aureus* GatCAB (PDB ID: 3IP4) was used as a template (Nakamura et al., 2010). To allow modeling of the fusion of GatC-GatA, the template primary sequence in the PDB file was renumbered such that GatC-GatA represented a single polypeptide. The output models were superposed to 1 mer of structure of the asparagine transamidosome from *Pseudomonas aerugino*sa (PDB ID: 4WJ3) (Suzuki et al., 2015) using UCSF Chimera software.

### RSPR Assays

These were performed as previously described (Zhu et al., 2018; Wang et al., 2020) with minor modifications. Briefly, for the RSPR plating assay, the strains of *M. smegmatis* were grown to OD ∼ 0.6–0.8. Bacteria were then plated on LB-agar or LB-rifampicin agar (25 μg/mL) and incubated at 37°C in the dark for 5 days, after which colonies were counted. Relative survival was calculated by the fraction survival on rifampicin-agar compared with non-selective medium. The fluorescence dilution assay was performed as before (Zhu et al., 2018) with one exception. Instead of Alexa-Fluor-488 as the fluorescent dye, NBD-amino-D-alanine (NADA) was used (any fluorescent dye that efficiently labels the cell-surface of the bacteria and would get diluted by growth and division can be used), otherwise, the protocol was similar as previously described. Flow cytometry was performed on a BD LSRFortessa cell analyzer and Flow-Jo software was used for analysis.

### Statistical Analysis

All experiments were performed at least twice on independent days with at least 3 biological replicates, with the exception of the Western blot, which was performed twice independently. Statistical analyses are described in the figure legends.

## Results

### A Modified Selection and Screen Strategy Identified Mutations in *gatC*, *gatA* and *gatB* as Mediators of Mycobacterial Mistranslation

**FIGURE 1:**
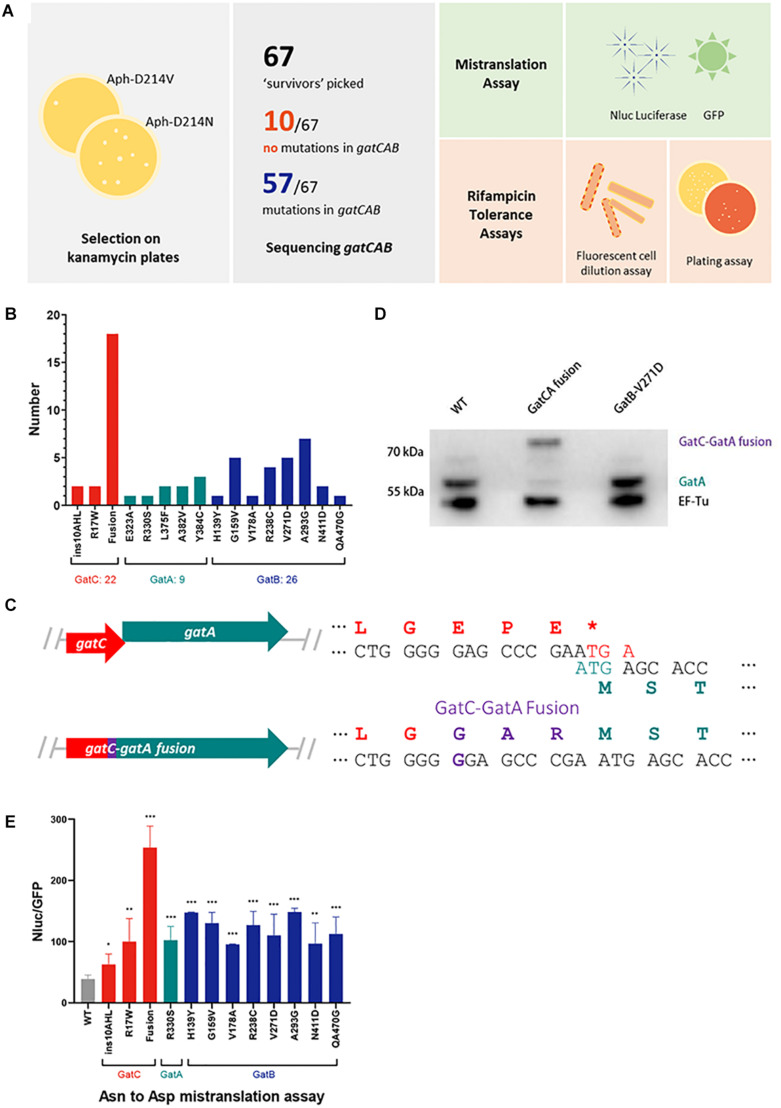
A forward genetic screen identifies a GatC-GatA fusion that mediates high rates of mistranslation. **(A)** Cartoon schematic of the selection strategy and subsequent phenotypic experiments. A reporter strain of *M. smegmatis* was plated on low-dose kanamycin agar to select for high mistranslation mutants (see section “Materials and Methods”). Serving strains had *gatCAB* genes sequenced. A selection of mutants were further characterized to measure mistranslation rates and rifampicin phenotypic resistance. **(B)** Relative frequency of mutants identified in the screen **(C)** Cartoon illustrating the mutation that abolished the stop codon of *gatC* and resulted in fusion of GatC-GatA. The wild-type genes are depicted in the top and the mutant below. **(D)** Western blot of GatA of wild-type, GatC-GatA fusion and a GatB mutated strains. EF-Tu as loading control. **(E)** Relative rates of asparagine to aspartate mistranslation using an Nluc/GFP reporter system (see section “Materials and Methods”). *p < 0.05, **p < 0.01, ***p < 0.001 by Student’s *t*-test.

We modified a previous selection and screen strategy that had been previously used to identify mutations in mycobacterial *gatA* as mediators of specific mistranslation (Su et al., 2016; Figure 1A). In the initial selection strategy, wild-type *M. smegmatis* was transformed with an episomal plasmid encoding a mutated kanamycin kinase gene, in which a critical aspartate residue necessary for enzymatic function (Boehr et al., 2001) was mutated to asparagine (Aph-D214N). Plating of strains on low-dose kanamycin-agar plates resulted in survival and growth of colonies of the reporter strain. This was due to the physiological and low-level (1–2%/codon) rates of mistranslation of asparagine to aspartate in mycobacteria, which would result in a proportion of functional kanamycin-kinase enzyme (Javid et al., 2014).

A concentration of kanamycin was selected in which at least 10-fold higher survival and growth of colonies was observed compared with a strain expressing a reporter plasmid with a valine substitution (Aph-D214V), which served as a control (see section “Materials and Methods”) to select for mutants with elevated rates of specific mistranslation targeting the indirect tRNA aminoacylation pathway (Javid et al., 2014; Su et al., 2016). In the prior forward genetic screen, survivors were then subjected to a further screen by transforming candidates with a reporter encoding a dual-luciferase mistranslation reporter (Kramer and Farabaugh, 2007; Chen et al., 2019). By contrast, for this screen we proceeded directly to sequence the *gatCAB* genes of all 67 mutant candidates (Figure 1A). Remarkably, 57/67 candidates had mutations in one of the three *gatCAB* genes (Table 1 and Figure 1B). Of particular note, the most commonly identified mutation (18/57, representing 31% of all mutants) was a single guanosine insertion in the 3′ end of the *gatC* gene, resulting in abolition of the *gatC* termination codon and predicted to result in a fused GatC-GatA polypeptide (Figure 1C). This mutation was identified in mutants selected from independent cultures, excluding a “jackpot” phenomenon being the sole reason for its over-representation among the mutants. To investigate whether the strain did indeed encode a fusion polypeptide, Western blot directed against GatA was performed. Unlike the WT strain, and a strain with a mutation in *gatB*, the “fusion” strain showed a ∼8 kDa shift in the size of GatA, verifying that the mutation did indeed result in a fused GatC-GatA protein (Figure 1D). A minor band, representing approximately <10% of the total intensity was visible at the normal mobility of GatA. This may have either represented a degradation product of the GatC-GatA fusion or initiation of GatA without GatC. Unfortunately, an antibody directed against GatC was not available to distinguish between these two possibilities.

**TABLE 1:** Description of mutations in *gatCAB* identified from the screen.

Gene	Mutation	Amino Acid Substitution
*gatC*	ins28GCCCACCTG	ins10AHL
*gatC*	C49T	R17W
*gatC*	ins290G	EPE97GAR + GatC-GatA fusion
*gatA*	A968C	E323A
*gatA*	C988A	R330S
*gatA*	C1123T	L375F
*gatA*	C1145T	A382V
*gatA*	A1151G	Y384C
*gatB*	C415T	H139Y
*gatB*	G476T	G159V
*gatB*	T533C	V178A
*gatB*	C712T	R238C
*gatB*	T812A	V271D
*gatB*	C878G	A293G
*gatB*	A1231G	N411D
*gatB*	CCAGGC1407GGG	QA470G

*All numbering is for the *M. smegmatis* genes.*

The relative mistranslation rates of 12 mutant strains representing mutations in all three *gatCAB* genes was measured using a gain-of-function Nluc/GFP reporter (Chaudhuri et al., 2018; Chen et al., 2019). All the mutant strains had elevated rates of asparagine to aspartate mistranslation, with the highest rate observed with the strain that contained the GatC-GatA fusion protein (Figure 1E). The relative lowest increase in mistranslation rate was observed in a mutant strain with a 9 bp in-frame insertion in the proximal part of the *gatC* gene (Figure 1E).

### Fusion of GatC-GatA Is Predicted to Radically Alter Enzyme Structure

**FIGURE 2:**
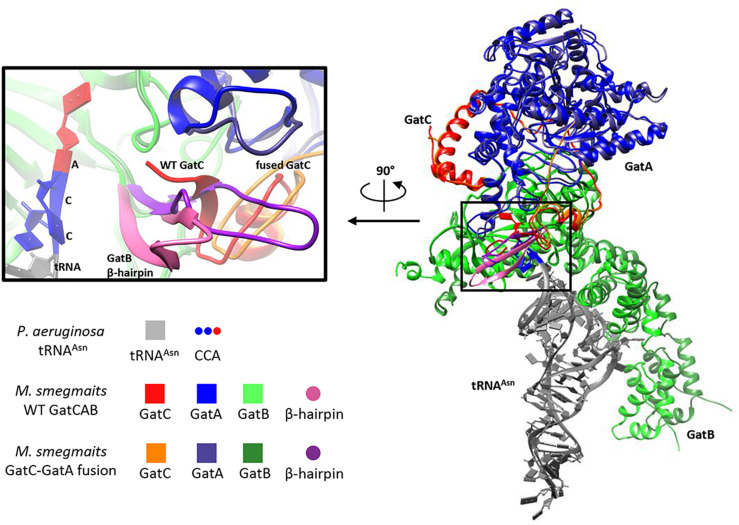
Modeling suggests GatC-GatA fusion disrupts GatCAB structure. Ribbon model of wild-type and GatC-GatA fusion *M. smegmatis* GatCAB and tRNA^Asn^ using Rosetta (see section “Materials and Methods”). The inset shows a close-up of the interaction between GatC, the beta-hairpin of GatB and the 3′-CCA of the tRNA. For clarity, the terminal amino acids ILGEPE of wild-type GatCA are not shown in the inset.

To model the potential perturbation of a fused GatC-GatA protein on enzyme structure, we constructed a model of the structure based on the solved structure of *Staphylococcus aureus* GatCAB (Nakamura et al., 2006) and see section “Materials and Methods.” In wild-type GatCAB, the β hairpin in the C-terminus of GatC interacts with the GatB Lys-146-Asp166 β hairpin (Figure 2, pink), resulting in a β sheet. When the C-terminus of GatC is fused to GatA, the β hairpin (Figure 2, purple), in GatB is no longer constrained in the former space, which may result in greater flexibility. This also leaves greater space between this β strand and the CCA-terminus of the tRNA^Asn^, which may affect the interaction between GatCAB and the aminoacyl-tRNA^Asn^.

### Mutations in *gatCAB* Generally Confer Increased Rifampicin Tolerance

**FIGURE 3:**
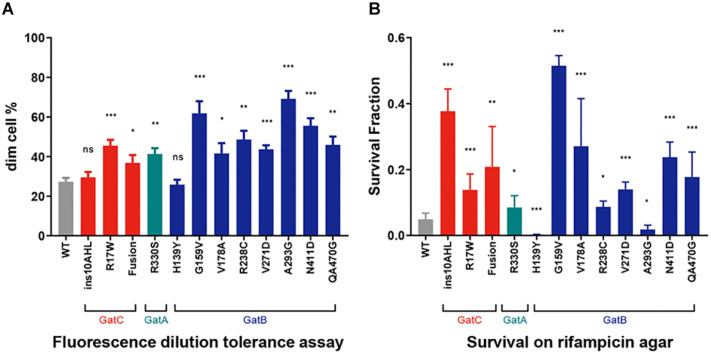
Increased Rifampicin-specific phenotypic resistance (RSPR) from GatCAB mutations. **(A)** Fluorescence-dilution assay: Wild-type and mutant strains of *M. smegmatis* were stained with NADA and grown in axenic culture + 50 μg/mL rifampicin overnight, and then loss of fluorescence measured by flow cytometry (see section “Materials and Methods”). **(B)** Wild-type and mutant strains of *M. smegmatis* were plated on 25 μg/mL rifampicin-agar and colonies counted after 5 days, compared with growth on LB agar alone. All experiments were performed with biological triplicates and data show mean ± SD. *p < 0.05, **p < 0.01, ***p < 0.001, ns *p* > 0.05 by Student’s *t*-test.

We had previously demonstrated that mutations in *gatA* that increase mistranslation rates also conferred increased tolerance to the first-line anti-tuberculous antibiotic rifampicin (Su et al., 2016). We performed two independent and complementary assays for rifampicin-specific phenotypic resistance (RSPR), a specific form of antibiotic tolerance that we had previously described in mycobacteria (Zhu et al., 2018). Of note, RSPR results in tolerance in growing mycobacteria, and is therefore distinct from non-replicating persistence. We first tested the strains with a fluorescence dilution assay we had previously developed (Zhu et al., 2018; Wang et al., 2020). In this assay, the cell wall of *M. smegmatis* is labeled with a fluorescent dye and then subject to growth in axenic culture ± bulk-lethal concentrations (concentrations that were bactericidal for >50% of the bacteria, the MIC_90_ for rifampicin in axenic culture was 5 μg/mL) of antibiotic. Tolerant bacteria that are able to not only survive, but also grow lose fluorescence by dilution due to cell division (Zhu et al., 2018). This assay measures growing-tolerant bacteria over 5 generations. All but two mutant strains, one harboring the in-frame insertion in GatC, and the other, GatB-H139Y, were more tolerant than WT *M. smegmatis* (Figure 3A). We then performed a second complementary assay for RSPR. Here, mycobacteria are plated on rifampicin-agar at a concentration (25 μg/mL) just above the plating MIC_90_ (20 μg/mL): tolerant bacteria are able to survive and grow on the bulk-lethal agar plate in the absence of *bona fide* rifampicin-resistance causing mutations in *rpoB* (Javid et al., 2014; Su et al., 2016; Zhu et al., 2018). This assay is a stringent test of antibiotic tolerance over the approximately 20 generations required to form a colony on an agar-plate. All the mutant strains resulted in increased rifampicin tolerance by this assay with the exception of two strains harboring mutations in *gatB* – GatB-H139Y and GatB-A293G. Surprisingly both appeared to be less tolerant than wild-type *M. smegmatis* (Figure 3B) in this assay.

## Discussion

In this study we have identified that mutations in any of the three constituents of the heterotrimeric enzyme GatCAB may result in elevated rates of specific mistranslation in *Mycobacterium tuberculosis*. Furthermore, the majority of mutations led to increased tolerance to the important antibiotic rifampicin, further confirming that mistranslation may play a potentially adaptive role under stress (De Pouplana et al., 2014; Mohler and Ibba, 2017; Evans et al., 2018).

A particularly striking finding from our study was the most frequently isolated mutation from the initial selection resulted in a fusion of the GatC and GatA proteins, GatC-GatA. This finding was confirmed by Western blotting, which showed that >90% of GatA was in the form of the fused polypeptide. A non-systematic, non-comprehensive analysis of approximately 500 *gatCAB* genes in different bacterial species failed to identify examples of a fused *gatC-gatA* single gene in other bacteria. Although the structure of mycobacterial GatCAB is not available, modeling suggested that the fusion would disrupt the normal structure of the enzyme (Figure 2). Despite this, the strain remained viable, albeit with altered translational fidelity. Western blotting identified a minor (∼10%) band of GatA, which may represent non-fused protein or degradation product. Prior studies on mycobacterial *gatA* mutations confirmed that strains with mutated *gatA* were viable, with increased mistranslation rates, despite approximately half wild-type GatA abundance (Su et al., 2016). Although we believe it is unlikely, it is therefore formally possible that the “fusion” strain was viable solely due to a small pool of wild-type GatA protein. However, even if alternative translation initiation allowed for translation of “wild-type” GatA at low abundance, it would not, in itself, allow translation of wild-type GatC, which entirely lacked a termination codon in the fusion mutant strain. Therefore, if the non-fused GatA is the only active form of the enzyme core, it would require functional viability of a heterodimeric “GatAB” enzyme, in the absence of GatC. Of note, all Archaea encode a dimeric GatDE enzyme that is employed exclusively in the indirect pathway for cognate aminoacylation of glutaminyl tRNA^Gln^ (Tumbula et al., 2000). However, GatDE is never involved in the aminoacylation of asparaginyl tRNA^Asn^. If the Archaeal species lacks AsnRS, the amidotransferase function is performed by a distinct, tRNA^Asn^-specific GatCAB enzyme (Tumbula et al., 2000). It would therefore be entirely without precedent for a dimeric amidotransferase enzyme being responsible for the indirect tRNA aminoacylation pathway for both glutaminyl and asparaginyl tRNAs. As such, we believe it is more likely that the functional enzyme involved the GatC-GatA fusion polypeptide.

Although most *gatCAB* mutations causing increased mistranslation also resulted in increased rifampicin tolerance, this correlation was not absolute. Only one mutation, GatB-H139Y, was not associated with increased rifampicin tolerance in either of the two assays we tested despite a robust increase in mistranslation. The reasons for this are not clear and will merit investigation in a future study. One potential explanation would be if there was a second-site mutation associated with increased rifampicin susceptibility (Wang et al., 2020) that negated the effects on RSPR of the mutation in *gatB.* Of potential greater interest, another mutation GatB-A293G was associated with *increased* tolerance in the fluorescence dilution assay, but *decreased* tolerance in the rifampicin-agar plating assay. Although both assays measure RSPR: tolerance in growing mycobacteria to rifampicin, they do have important differences. The fluorescence dilution assay interrogates growth tolerance over a short period (16 h) representing approximately 5 generation times for *M. smegmatis* (Zhu et al., 2018), but at much higher (10×) concentration of drug compared with the MIC. By contrast, the plate assay requires tolerant bacteria to survive and grow over 20 divisions to form visible colonies (Zhu et al., 2018), but on relatively lower MIC (1.2×). The liquid versus sold medium also represents another difference between the two assays, of potentially unknown significance. We recently described that mycobacterial RSPR involves two distinct phases of antibiotic tolerance. Initial exposure to rifampicin results in tolerance that may be mediated, for example, by mistranslation or by basal upregulation of RpoB-RpoC (Zhu et al., 2018). However, rifampicin exposure itself causes paradoxical upregulation of RpoB-RpoC, which in turn increases phenotypic resistance to the drug and allows survival and growth in otherwise lethal rifampicin concentrations (Zhu et al., 2018). It should be noted that despite the ability to grow in bulk-lethal rifampicin-containing media, both mistranslation-mediated RSPR and rifampicin-mediated upregulation of RpoB-RpoC are examples of phenotypic, not *bona fide* genetic resistance (Javid et al., 2014; Su et al., 2016; Zhu et al., 2018). This is not due to, for example, to a change in the MIC, even when caused by mutations that increase the proportion of the tolerant subpopulation (Su et al., 2016; Wang et al., 2020). We recently described certain mutations that led to increased basal rifampicin tolerance, but decreased tolerance to rifampicin re-exposure (Wang et al., 2020). It is therefore conceivable that the GatB-A293G mutation may also fall into this category, and further studies will be required to investigate the observed phenomenon. Although our experiments were performed in *M. smegmatis*, isolates with mutations in all three *gatCAB* genes occur in clinical strains (Su et al., 2016). Our work suggests that many of these may have clinically relevant phenotypes such as increased antibiotic tolerance.

More generally, our work highlights that overall, the indirect tRNA aminoacylation pathway contributes to adaptive mistranslation in mycobacteria, and that disruption of any of the three constituent members of the essential GatCAB enzyme may decrease the fidelity functions of this pathway. Our study also confirms the remarkable resilience of mycobacteria to perturbations in specific translational fidelity as demonstrated by a potential radical alteration of structure in a central, essential enzyme in the indirect tRNA aminoacylation pathway.

## Data Availability Statement

The raw data supporting the conclusions of this article will be made available by the authors, without undue reservation.

## Author Contributions

BJ conceived of the project. BJ, R-JC, and H-WS designed the research. R-JC performed the majority of experiments with some assistance by Y-YL. BJ wrote the manuscript with input from R-JC and the other authors. All authors contributed to the article and approved the submitted version.

## Conflict of Interest

The authors declare that the research was conducted in the absence of any commercial or financial relationships that could be construed as a potential conflict of interest.
